# Nanoscale Elasto-Capillarity in the Graphene–Water System under Tension: Revisiting the Assumption of a Constant Wetting Angle

**DOI:** 10.1021/acs.langmuir.3c01259

**Published:** 2023-08-25

**Authors:** Movaffaq Kateb, Andreas Isacsson

**Affiliations:** Department of Physics, Chalmers University of Technology, SE-412 96 Gothenburg, Sweden

## Abstract

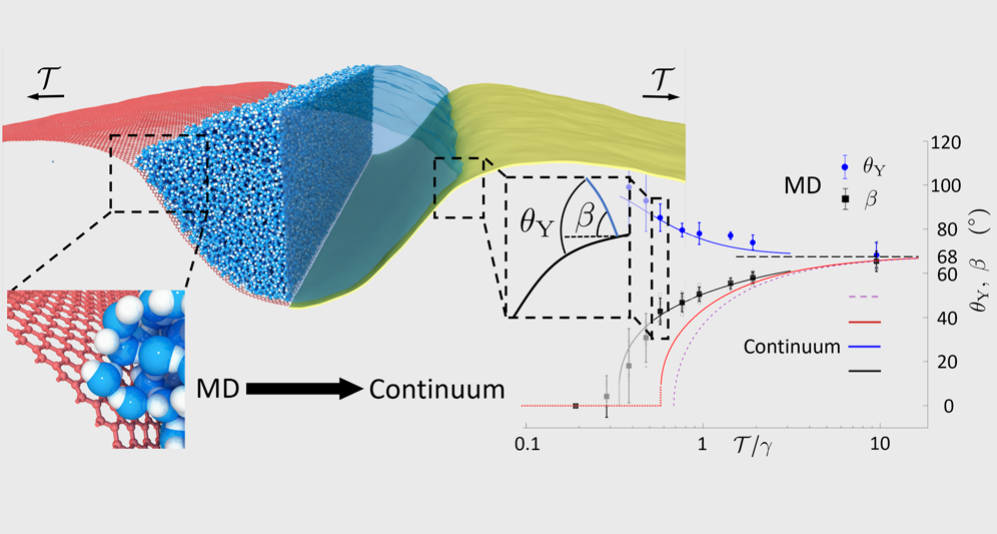

Wetting highly compliant surfaces can cause them to deform. Atomically thin materials, such as graphene, can have exceptionally small bending rigidities, leading to elasto-capillary lengths of a few nanometers. Using large-scale molecular dynamics (MD), we have studied the wetting and deformation of graphene due to nanometer-sized water droplets, focusing on the wetting angle near the vesicle transition. Recent continuum theories for wetting of flexible membranes reproduce our MD results qualitatively well. However, we find that when the curvature is large at the triple-phase contact line, the wetting angle increases with decreasing tension. This is in contrast to existing macroscopic theories but can be amended by allowing for a variable wetting angle.

## Introduction

**Figure 1:**
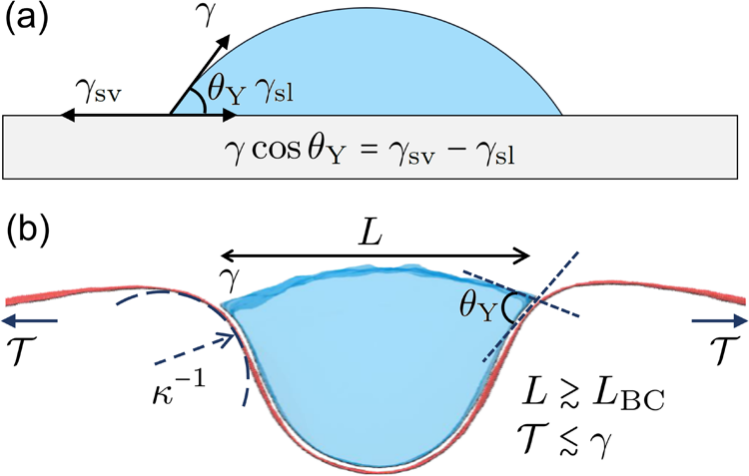
(a) Wetting of a flat and rigid substrate. The wetting angle θ_Y_ is obtained via balancing in-plane components of the liquid–vapor (γ), the solid–vapor (γ_sv_), and the solid–liquid (γ_sl_) surface tensions (free energy densities), at the triple-phase contact line (TCL). (b) Wetting-induced deformation for a water nanodroplet on graphene under tension  obtained from molecular dynamics. In the vicinity of the TCL, when , the graphene bends smoothly with a curvature κ of the order of *L*_BC_^–1^. Here, all of the force components must be balanced.

The Young–Dupré equation determines the wetting angle θ_Y_ of liquid droplets resting on rigid flat substrates. It can be derived via balancing in-plane surface tensions at the triple-phase contact line (TCL)^1,2^ (see Figure 1a). It has been used to study a variety of systems under different conditions and size scales, ranging from self-cleaning surfaces,^3^ deposition and painting^4^ at ambient temperatures, as well as epitaxial growth^5^ and welding^6,7^ at high temperatures. However, several disciplines within nanotechnology are increasingly exploiting thin elastic structures in combination with wetting for precise structural control, drug delivery, or sensing. Other systems rely on biomimetic designs whose functionality draws on the interplay between surface tension and deformations. In these cases, Young’s treatment must be modified.

The first to shed light on wetting-induced deformations of the substrate, i.e., elasto-capillarity, was probably Lester.^8^ This requires balancing the tension components normal to the surface. The development and interest in elasto-capillary phenomena have increased in recent years. Thanks to advances in imaging techniques, our understanding of elasto-capillary effects in, for instance, soft bulk matter has matured.^9−11^ Recently, it has also found applications in coating of polymers, adhesives, and bioinspired designs,^12−15^ micro- and nanofabrication,^16,17^ bending of fibers,^18^ or nanowire arrays.^19^ It has further been shown to be beneficial for capillary origami, i.e., wrapping thin solids around droplets^20,21^ or bubbles.^22,23^

For elasto-capillary effects to be relevant, the linear size of the droplet, *L*, should be comparable to the elasto-capillary length. For bulk solids, this length is set by *L*_EC_ = γ/*E*, where *E* is the substrate Young’s modulus and γ the liquid–vapor surface tension. This definition has been successfully applied to biomembranes and soft matter systems.^24−27^ However, in thin membranes, *L*_EC_ can be significantly smaller than the interatomic distances.^28−30^ Despite having a large *E*, these may still bend easily. The relevant length scale is then *L*_BC_ = (*B*/γ)^1/2^, where *B* is the bending rigidity of the membrane. Physically, *L*_BC_ can be interpreted in terms of the inverse curvature of the membrane at the TCL^24^ (see Figure 1b). While ample research has been done on wetting of thin membranes, investigations on how these phenomena carry over to the nanoscale are lacking.

Probing membrane elasto-capillarity on the nanoscale requires a material with extremely small bending rigidity. For water on graphene (*B* ≈ 2 eV, γ = 72 mN/m), one obtains *L*_BC_ ≈ 2 nm, making graphene an optimal choice. Imaging of droplets on suspended graphene has already been reported,^31^ the most recent with droplet diameters down to a micron.^32,33^ In fact, in ref (33), droplets with diameters down to 200 nm are clearly visible. Although wetting of graphene has been studied extensively, both theoretically and experimentally, wetting-induced deformations have received less attention. In particular, the dependence on the nanoscale structure near the TCL under external tension  has not been studied. While *L* ≳ *L*_BC_ is necessary for deformation, under high tension (), this deformation will still be small. For instance, the suspended membranes in experiments^31−33^ have been under too large tensions for elasto-capillary effects to be visible. When , the situation in the vicinity of the TCL is more subtle,^34−37^ and care must be taken with the length scales when deformations and θ_Y_ are measured.

A recently addressed issue is how the tension and the wetting angle change across the TCL, and how this affects the details of the local morphology in inextensible membranes. Neukirch et al.^38^ used a two-dimensional (2D) variational approach, and Schulman and Dalnoki-Veress^34^ showed that in a suspended membrane,  contributes to the force balance at the TCL. Note that the wetting angle θ_Y_ in refs (38) and (34) was assumed to be independent of  when measured locally at the TCL. This does not necessarily imply that the droplets remain spherical for membranes with nonisotropic tension.^39−41^ On the experimental side, Kumar et al.^36^ recently demonstrated an experimental technique to directly probe the variation in membrane tension across the TCL. Still, direct measurements of θ_Y_, and depicting the details of the contact line at the nanoscale, remain out of reach.

Molecular dynamics (MD) provides a unique tool to probe the TCL on the atomistic scale.^42,43^ To investigate deformations at the TCL in a membrane under tension, we have performed large-scale MD simulations on a water–graphene system and compared the results to recent macroscopic continuum theories for a cylindrical droplet on an infinitely wide membrane. This geometry is common in MD studies of surface wetting for several reasons.^44−51^ First, it requires fewer atoms in the fluid phase, making it computationally less expensive than a full three-dimensional (3D) treatment. Second, it avoids problems arising from contact-line curvature.^52,53^ Finally, a 2D model allows us to isolate the effects of tension and focus on the behavior of the wetting angle, as the Gaussian curvature is zero. This is in contrast to 3D models, where wrinkling can obscure the underlying physics. For the graphene–water system, existing MD studies mostly treat graphene to be rigid and flat (cf. refs (49,50) and (53)). While some considered the interplay between wetting and deformations,^20,21,54^ comparisons between continuum models and large-scale atomistic simulations have not been done before.

Using MD puts demands on droplet size, relaxation times, and choice of interatomic force fields.^55^ To fulfill *L* ≳ *L*_BC_, we have simulated systems with up to 1 order of magnitude more molecules in the fluid (3.5 × 10^5^) than what is common in the literature (≲10^4^). Importantly, this size also prevents thermal agitation by flexural modes from dominating the dynamics.^54,56^ To ensure that we reach a stable thermal equilibrium state, our simulations were relaxed for 4–20 ns. This is considerably longer than used for studying wetting of rigid graphene (0.1–1 ns).

## Methods

Classical MD simulations were performed using LAMMPS^57^ (http://lammps.sandia.gov/) (version March 3, 2020). To avoid manually tuning solid–liquid interactions,^20,21,54^ we employ all atomic-optimized potentials for liquid simulations (OPLS-AA)^58,59^ for graphene. For the water molecules, we use the rigid extended-point-charge (SPC/E) model,^59^ which is compatible with OPLS-AA for large organic molecules.^60^ Postprocessing of data and graphical representations were done using the OVITO package (http://ovito.org/).^61^

Within OPLS-AA, the graphene–water interaction is treated via a Lennard-Jones (LJ) potential. There have been many efforts to find LJ parameters for the interaction between water and rigid graphene. These studies reached values that are close to that of OPLS-AA with SPC/E (cf. refs (49) and (51)). In our simulations, we used a cutoff of 12 Å for short-ranged vdW and Coulomb interactions, while long-ranged electrostatic forces were solved in k-space using the particle–particle particle-mesh (PPPM) method. Here we used an accuracy of 1 × 10^–5^ kcal/(mol Å) for the production runs. We did not observe any considerable differences using increased accuracy.

The velocity Verlet^62^ algorithm was employed for integrating the equations of motion, using a time step of 2 fs. A Langevin thermostat (*T* = 300 K) was used for temperature control of water with 2 ps damping. Thus, we treat water in the canonical (NVT) ensemble. The graphene was thermostated every 2 ps using a Nosé–Hoover thermostat, where we adjusted the box dimension in the transverse direction along the width (*z*), in order to relax stresses, as measured via a Nosé–Hoover barostat. This produces samples from the isothermal–isobaric ensemble (NPT) for graphene. The initial velocities of all atoms were drawn from a Gaussian distribution corresponding to *T* = 300 K. This way of thermostatting avoids the “flying ice-cube” artifact,^63^ where the thermostat gradually removes energy from high-frequency modes and adds it to low-frequency ones. For small amounts of water, an alternative is to only thermostat the graphene and not the droplet (cf. ref (54)).

Our samples ranged from 100 to 250 nm in length (*x*-direction) and 3 to 12 nm in width (*z*-direction), with a C–C distance of 1.4148 Å prior to relaxation. Periodic boundary conditions were used in the transverse direction (*z*-direction). To impose an external tension , forces in the *x*-direction along the ribbon were uniformly distributed over two small regions, one at each edge. These regions extended a distance of 2 nm from the edges into the ribbon. The rest of the ribbon was allowed to interact freely with the water. Normal to graphene, we inserted enough vacuum to make self-interactions negligible. These boundary conditions allow the formation of a stable hemicylindrical water droplet with an axis of symmetry parallel to the *z*-axis.^46,49^ All simulations used the same ratio between the water volume and graphene surface area. For 100, 150, 200, and 250 nm long and 12 nm wide ribbons, we consequently had hemicylindrical droplets approximately 20, 24, 28, and 31 nm in diameter, respectively. This amounts to between 53 × 10^3^ and 133 × 10^3^ water molecules.

There are two common approaches for determining the wetting angle in MD. Conventionally, the positions of the atoms and molecules in the liquid phase are averaged over a specific time to obtain the average density distribution. This is followed by fitting the outer water layer, or a contour of constant average density close to the surface, to a circular contour. The main reason for this averaging is to minimize errors associated with the change of instantaneous angles. These can arise due to the high mobility of small droplets, where one must correct for the drift of the center of mass during averaging. In the second approach, which works for relatively large droplets (≥10^5^ atoms), no time averaging is done, allowing also dynamic wetting angles^64^ to be determined.

**Figure 2:**
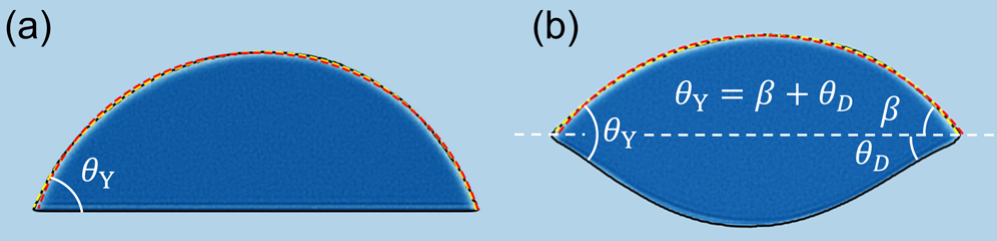
Fitting of wetting angles with respect to molecular dynamics data. The local density was determined on a 200 × 200 grid by time averaging after relaxation. (a) Determination of the wetting angle θ_Y_ on a flat substrate. By fitting a circular contour to the water, θ_Y_ can be determined. (b) For the deformed case (partial wetting), better fits were obtained by using an elliptic contour. Two angles were extracted, β and θ_D_, where θ_Y_ = β + θ_D_.

**Figure 3:**
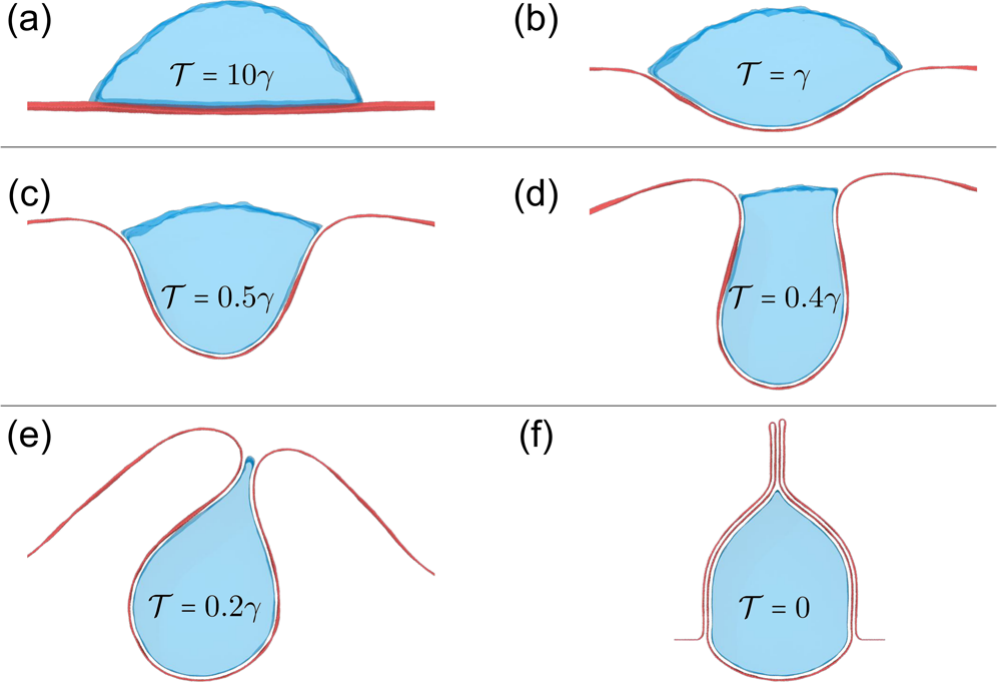
Equilibrium shapes of the graphene water system for different amounts of external applied tension  obtained from molecular dynamics on a 100 × 12 nm^2^ graphene ribbon. Only the portion of the ribbons in the vicinity of the droplet is shown. (a) High external tension. The graphene sheet is only slightly buckled, and the water surface has a circular profile. (b, c) Intermediate tensions. Decreasing  induces a gradually larger deformation, and causes the water surface to attain a slightly elliptical shape. (d, e) Shapes in the vicinity of the vesicle transition. Here, low-frequency thermal motion may rock the entire vesicle, as seen in (e). (f) Vesicle state. In the absence of external tension, a complete vesicle is formed. For cases (a–d), it is clear that as the transition is approached, the wetting angle θ_Y_ increases.

To determine the wetting angle at the triple-phase contact line (TCL), we relaxed the structures for at least 4 ns. However, most systems required much longer relaxation times, occasionally well over 20 ns, depending on size and tension. In particular, close to the vesicle transition (see Figure 3d,e), long relaxation times were needed. We then calculated the number of water molecules within a 200 × 200 square mesh in the *xy*-plane (integrating over *z*) and averaged over 1 ns to obtain the water density profile (Figure 2). Least square fitting was then used to fit either a circle or an ellipse to obtain the contour of the water surface. As our droplets are very large, the exact density contour chosen is not critical, as long as it is close to the surface (see Figure 2). As seen in Figure 2b, we could not get an optimal fit using circular contours in the partial wetting case when the graphene is deformed. Instead, we fitted the density profile with an ellipse which provides a better fit. As a result, we obtain values of β (see Figure 2), slightly smaller than those fitting with a circle.

Using SPC/E with OPLS-AA resulted in a wetting angle of θ_Y_ = 68.5° for a flat water–graphene interface (Figure 2a). While the exact wetting angle of water on suspended graphene is still under debate (see refs (31−33)), we note that although SPC/E can provide a reasonable value^65^ for the surface tension (γ ≈ 70.2 ± 2.1 mN/m), it can underpredict γ when using PPPM,^66^ giving a value as low as γ ≈ 55.4 mN/m. To benchmark our results for the wetting angle, we extensively verified different aspects of our simulation with the existing literature. In particular, with regard to the wetting angle, we accurately reproduced the results of ref (49).

Here, the exact value of θ_Y_ is less important as we study the applicability of continuum theories on the nanoscale and the variation of the wetting angle under deformation.

## Results and Discussion

Figure 3 shows representative MD results from a 100 × 12 nm^2^ graphene ribbon. The overall behavior, including orders of magnitude for parameters (see below), confirms the theoretical predictions of the continuum theory of Kozyreff et al.^37^ In particular, our simulations clearly reproduce the transition to a vesicle-like state below a critical value of the applied external tension . However, as can already be discerned from the shapes in Figure 3, the wetting angle increases when decreasing .

Note that Figure 3 shows snapshots of the systems. To fully appreciate the situation, dynamics must also be taken into account. For instance, determination of the angles β and θ_D_ involves averaging over several hundreds of snapshots, with subsequent fitting (e.g., Figure 2). For very large deformations, low-frequency oscillations are present. In these, the part encapsulating the water ”swings”, tilting the system from side to side, or shows behavior reminiscent of breathing modes. This is partly visible in panels (d) and (e). To compensate for this, we ensured that the symmetry axis was always rotated to a vertical position before the angles were determined. For the situation in panel (f), we define the angle β as zero, while θ_Y_ is undefined. Here, the van der Waals interactions between different parts of the sheet will determine the final shape rather than the surface tension. Further, the formation of sharp folds, as seen in panel (f), may also lead to bond breaking. Being a nonreactive force field, such phenomena cannot be studied using OPLS-AA.

**Figure 4:**
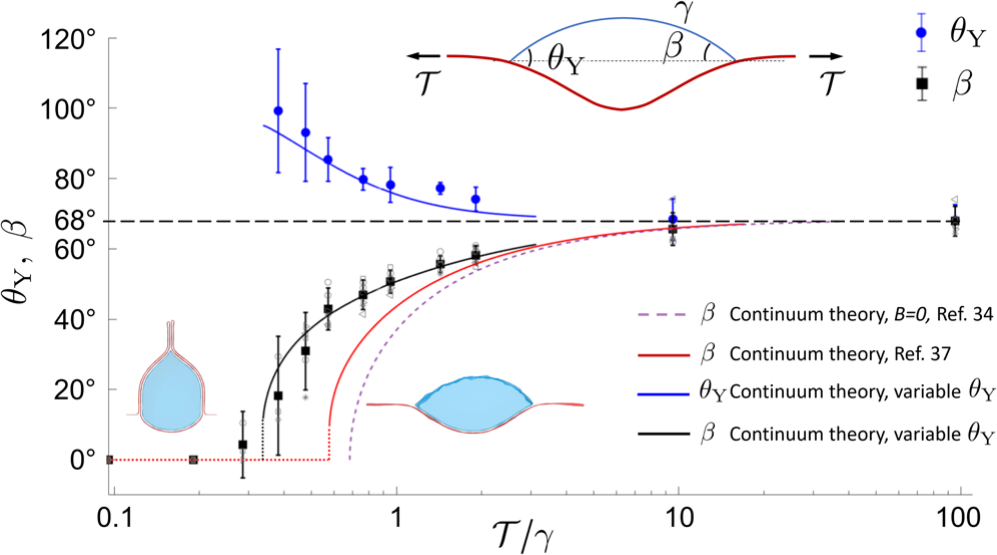
Dependence on wetting angle θ_Y_ on external tension  obtained from MD. The average wetting angle θ_Y_ and average angle β were calculated from data obtained on samples ranging from 100–250 nm long and 3–12 nm wide with the number of water molecules scaled accordingly. The wetting angle (blue circles) increases with decreasing tension. The solid red line is obtained from the continuum theory with a fixed θ_Y_ = 68.5°. The solid blue and black lines result from continuum theory using a variable θ_Y_. The dashed purple line results from the superflexible continuum model in eq 2. The transition to the vesicle state occurs when β tends to zero.

Our main findings are well summarized in Figure 4, showing the wetting angles obtained from MD simulations along with the angle β. The data points are averages over samples of sizes 100 × 12, 150 × 3, 150 × 6, 200 × 6, 200 × 12, and 250 × 12 nm^2^, with error bars three times the standard deviation in the mean. From the data set, we have excluded some points very close to the vesicle transition where it was not possible to extract the angles due to the very narrow constriction (Figure 3e). The increased noise in the data upon approaching the transition is signaled by the larger error bars in Figure 4. Data from some of the largest samples that underwent transverse wrinkling during the relaxation process, leading to metastable solutions, were also discarded.

There is one expected and one unexpected result in Figure 4. As expected, for low tensions, there is a transition to a vesicle state below a critical tension . The exact value of the critical tension cannot be discerned, as the noise in the data increases close to the transition. However, and more important, is that we see a clear and significant dependence on the wetting angle θ_Y_ with decreasing tension, which we cannot attribute to numerical error. This is in contrast to the predicted and measured constancy of the wetting angle with changing external tension in macroscopic systems.

For a droplet of linear size *L* ≫ *L*_BC_ it is possible to treat graphene as superflexible (*B* → 0) when looking at length scales ∼ *L*. For this special case, Schulman and Dalnoki-Veress^34^ found that using a macroscopic constant wetting angle θ_Ym_, and assuming a sharp kink at the TCL, experiments could be fitted using force balancing provided that the parallel traction *n*_∥_ at the TCL obeyed the boundary condition
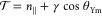
1Under this assumption, they showed that the macroscopic angle β_m_, assuming that θ_Ym_ remains constant, obeys the relation
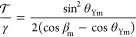
2implicitly providing β_m_ as a function of the scaled tension . It is important to note here that β_m_ and θ_Ym_ are macroscopic angles. They are thus not necessarily the same as β and θ_Y_, which we here define on a microscopic level, where the membrane curvature changes smoothly across the TCL. The result of using eq 2 is shown as the dashed purple line in Figure 4.

Equations 1 and 2, which seemed valid for fitting macroscopic experiments,^34^ were later derived using variational approaches,^37,38^ accounting also for finite *B*. However, also the theory of Kozyreff et al.^37^ (shown as the solid red line in Figure 4) still deviates significantly from our MD results near the vesicle transition, and we need to adjust the model. To understand the reason for this, it is necessary to briefly recapitulate the ingredients and underlying assumptions of this theory and how it differs from MD.

**Figure 5:**
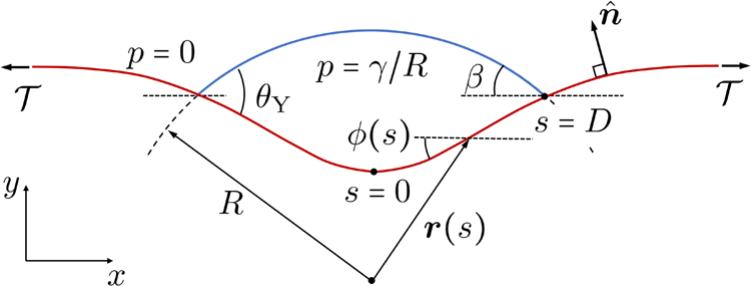
Geometry for the continuum treatment (adapted from ref (37)). An infinitely long membrane of width *W*, allowed to displace only in the *xy*-plane, is characterized by the radius vector ***r***(*s*) lying in the *xy*-plane. The parameter *s* is 0 on the symmetry axis and *s* = *D* at the TCL. The radius of curvature of the water droplet is *R*. Shown are also the angles θ_Y_, β, and ϕ(*s*).

The model considers a rectangular graphene ribbon of finite width *W* and total length *L*_0_ under tension . A cross section of the geometry is shown in Figure 5. We assume a symmetric situation, with the water surface having a circular cross section with radius *R*. To find the differential equations that govern the shape of the membrane under partial wetting, we minimize the free energy *F* = *F*_el_ + *F*_w_, where *F*_el_ is the elastic free energy and *F*_w_ contains all contributions from the water droplet including the surface free energies.

The graphene surface can be parametrized by a curve described by radius vector ***r***(*s*) lying in the *xy*-plane. In terms of ***r***(*s*), the elastic free energy for a sheet of width *W* is, in the inextensible case, given by^67^
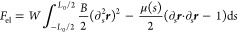
3Here, *B* is the membrane bending rigidity and μ is a Lagrange-multiplier ensuring that *s* is the proper arc length. The parametrization is chosen such that *s* = 0 corresponds to the symmetry axis of the deformed membrane and *s* = ± *D* at the TCL (see Figure 5).

The free energy of the water droplet, containing *N* particles interacting with the sheet, is determined by
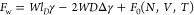
4Here, *Wl*_*D*_ is the area of the water–vapor interface, *WD* the area of the water–graphene interface, and Δ*γ* = γ_sv_ – γ_sl_ is the difference between the surface free energy densities of the graphene–vapor interface and the water–graphene interface. The term *F*_0_(*N*, *V*, *T*) in eq 4 is the free energy for the water droplet of volume *V* = *WA*, where the cross-sectional area *A* depends on ***r***(*s*), *D*, and β. In accordance with the MD simulations, we here assume the droplet to be surrounded by vacuum and that all water molecules are contained in the droplet.

For a (symmetric) partial wetting state given by a fixed ***r***(*s*), minimizing *F* with respect to *D* and β, and making use of *p* = −∂_*V*_|_*N,T*_*F*_0_, reproduces the Laplace pressure *p* = γ/*R* for a hemicylindrical droplet and the Young–Dupré equation γcos θ_Y_ = γ cos(β + θ_D_) = Δ*γ* for the wetting angle at the TCL. Hence, unless γ/Δ*γ* changes with tension or local curvature, it follows that θ_Y_ should remain constant.

To obtain the differential equations for the shape of the sheet, we vary *F* with respect to ***r***(*s*) to obtain the pressurized elastica
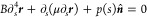
5Here, *p*(*s*) = γ/*R* for |*s*| ≤ *D* and zero outside the droplet. The vector ***n̂*** is the unit normal to the graphene surface. Using the Frenet–Serret formulas, and projecting (eq 5) onto the surface normal and tangent, respectively, μ(*s*) can be solved for in terms of the curvature κ(*s*) and the exernal tension . This gives , leaving a single equation for the curvature
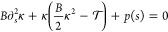
6

From eq 6, the ODE system in ref (37) is readily reproduced by identifying the tractions  and *n*_⊥_ ≡ – *B*∂_*s*_κ, i.e.,
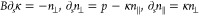
7To align the orientation of the system, we introduce the angle ϕ between the curve and the *x*-axis (see Figure 5), which gives one more equation, ∂_*s*_ϕ = κ.

We solve the system (eq 7) numerically with the initial conditions ϕ(*s* = 0) = *n*_⊥_(*s* = 0) = 0, and vary the remaining free parameters to match the boundary conditions at the TCL (*s* = *D*)
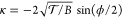
8
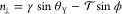
9
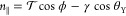
10under the constraint that the cross-sectional area *A* remains constant. Condition 8 follows from the assumption of an infinite sheet where ϕ(∞) = κ(∞) = 0 and that the tension is acting only along the *x*-axis. Conditions 9 and 10 ensure force balance at the TCL, while requring a constant *A* amounts to treating the fluid as incompressible. Finally, we use *p* = γ/*R* and treat *R* as an adjustable parameter.

To compare our MD results to the continuum theory in eq 7 requires numerical values for *A*, *B*, γ, and θ_Y_. While we have established values of γ and θ_Y_ (see the Methods section) and know the cross section *A* from our simulations, the bending rigidity *B* must be determined independently. Existing experiments and theories estimate *B* to a few eV. However, thermal out-of-plane fluctuations will affect *B* and introduce a wavelength dependence.^68^ To find the value of *B* that corresponds to the finite temperature continuum limit and is valid for the relevant radii of curvature in our simulations, we have simulated pure bending of ribbons at *T* = 300 K.

**Figure 6:**
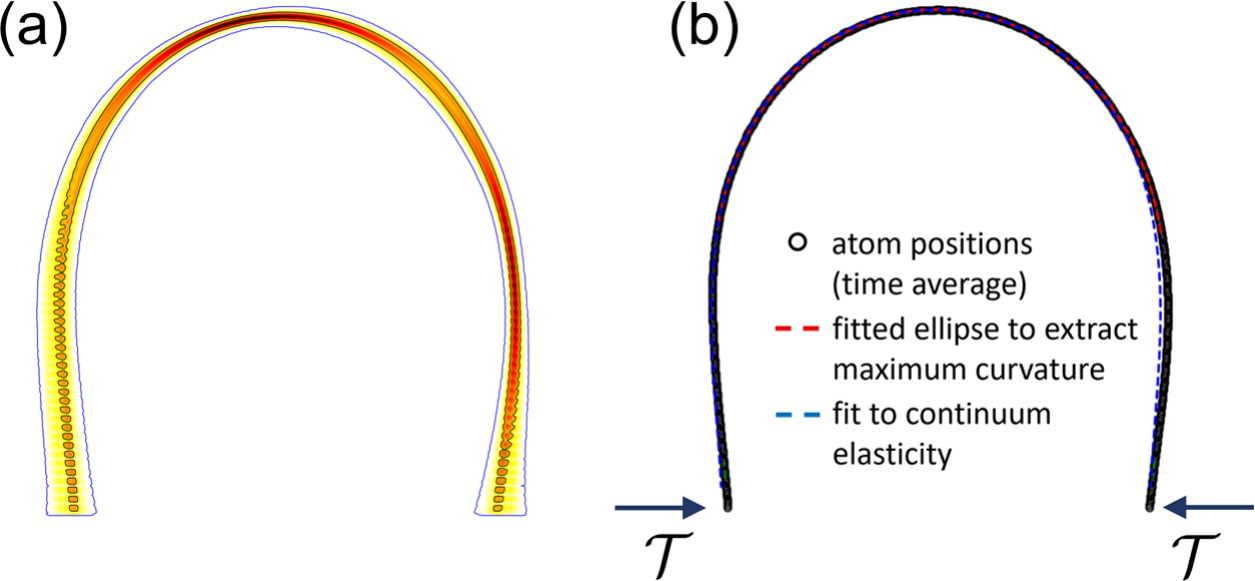
Comparison and fitting of MD results to continuum elastic theory to extract the bending rigidity. Using MD, graphene nanoribbons were subjected to compressive loads  at the edges (*T* = 300 K). The resulting shapes were fitted to continuum elastic theory to extract bending rigidity *B*. (a) Probability density, obtained from MD, for atom positions of a graphene nanoribbon (80 × 12 nm^2^) subject to compressive load  at the end points. (b) Average positions of the atoms (black circles) and fitting to continuum elastic theory. To fit the shape, the curvature at the top was extracted (red dashed line), and then the overall shape was fitted (blue dashed line).

The renormalized values of *B* for OPLS-AA were extracted by applying different amounts of compressive stress to edges of graphene nanoribbons with lengths between 50 and 200 nm at *T* = 300 K. After relaxation, we fitted the ribbon shapes to the corresponding solutions of eq 5 with *p* = 0, as shown in Figure 6. For ribbons with lengths exceeding 50 nm, we consistently obtained values in the range *B* ∼ 2.5–3.5 eV, with lower values for shorter ribbons. The uncertainty comes from the large thermal fluctuations, as can be seen in Figure 6a, where the probability density for the atomic positions is shown for one sample. As shown in Figure 6b, we can get excellent fits considering graphene as an inextensible continuum membrane.

For the sake of completeness, we also extracted the 2D elastic modulus by applying in-plane tensile stress to ribbons. Here, OPLS-AA predicts *E* ≈ 340 N/m, consistent with other data in the literature. We have also obtained the dispersion relations for flexural phonons (ZA) within the OPLS-AA, as well as the renormalized bending rigidity for nearly flat sheets by calculating the height–height correlation function. Also here, we find excellent agreement with the literature (see, for instance, Figure 2 in ref (68)).

The red curve in Figure 4 shows the predicted angle β from the continuum model by using the extracted parameters. As can be seen, it fits relatively well on a qualitative level and also gives a correct order of magnitude estimate. Rescaling the continuum equations, it can be shown that β depends on only three dimensionless quantities. It can be written as , where Γ = γ*A*/*B*. For a constant value of θ_Y_, smaller values of Γ will push the vesicle transition to lower values of . For the red curve in Figure 4, we used upper bounds on *B* and lower bounds on γ and *A*, resulting in a lower bound of Γ = 29. Still, the continuum theory predicts a transition to the vesicle state at . This is considerably larger than the value observed from MD ().

Further, the discrete data points in Figure 4 correspond to cross-sectional areas ranging from 250 to 628 nm^2^. Thus, it may seem surprising that most data points seem to fall on the same curve as this changes Γ by a factor of 2.5. This robustness to changes in Γ is also present in continuum theory, which predicts that Γ must change by an order of magnitude for the shift in the curve to be comparable to the uncertainty in the angles β and θ_Y_. Hence, the discrepancy between MD and continuum theory cannot be attributed to uncertainties in the parameters *A*, γ, or *B*. As we observe a nonconstant wetting angle (blue circles in Figure 4), we instead look to amending the continuum theory to accommodate a varying θ_Y_.

By relaxing the assumption that the wetting angle should remain constant for nanoscale droplets, it is still possible to use the continuum theory provided by the pressurized elastica (eq 5).

We do this by changing θ_Y_ in the boundary conditions 8–10 to obtain better agreement with the observed β from MD simulations. There is, however, no unique functional form that can be applied to perform, e.g., a least-squares fit directly to β or θ_Y_. Instead, we use a simple Lorentzian trial function for the variation of θ_Y_, to obtain the best fit for β. This choice of trial function is sufficient to clearly highlight that a variable θ_Y_ makes the predicted β in better agreement with the MD data. The results of this procedure are shown as solid blue and black lines in Figure 4. As seen in Figure 4, this treatment works well, lending support to a nonconstant wetting angle, but also that we are using a large enough system for a continuum treatment to be meaningful.

One should note here that this amended continuum theory cannot fully account for the behavior seen in Figure 3e,f. Here, two parts of the graphene sheet come in such close proximity to each other that the vdW interactions between the carbon atoms become important. This is most extreme in the case of Figure 3f, where the vdW interactions between carbon atoms cause the sheet to fold on itself. At these high curvatures, the continuum theory of the graphene sheet will also start to break down.

The physical origin of the changing wetting angle is elusive. To rule out that θ_Y_ depends on the local tension at the TCL, we first simulated flat membranes under varying amounts of external tension  and found the wetting angle to be constant. There are, however, several other properties in the MD simulations that are absent in continuum theory.

For a flexible membrane, the deformation is directly connected to the local values of γ and *p*, which will not be uniform within the droplet^69^ and subject to thermal fluctuations. The continuum theory assumes *p* and γ to be constant, uniform, macroscopic thermodynamic averages, related via *p* = γ/*R*. Thus, one can expect departures from macroscopic theory, in particular for large deformations. Furthermore, on the nanoscale, one must account for the TCL having an effective finite width. This stems from the interatomic forces between the water and graphene being distributed over distances of approximately 1 nm. The membrane curvature at the TCL on this length scale is not constant (in contrast to a flat membrane), which could influence our measured θ_Y_. This is in contrast to the continuum theory, where the surface tension exerts a force acting along a line of zero width, predicting a constant wetting angle.

**Figure 7:**
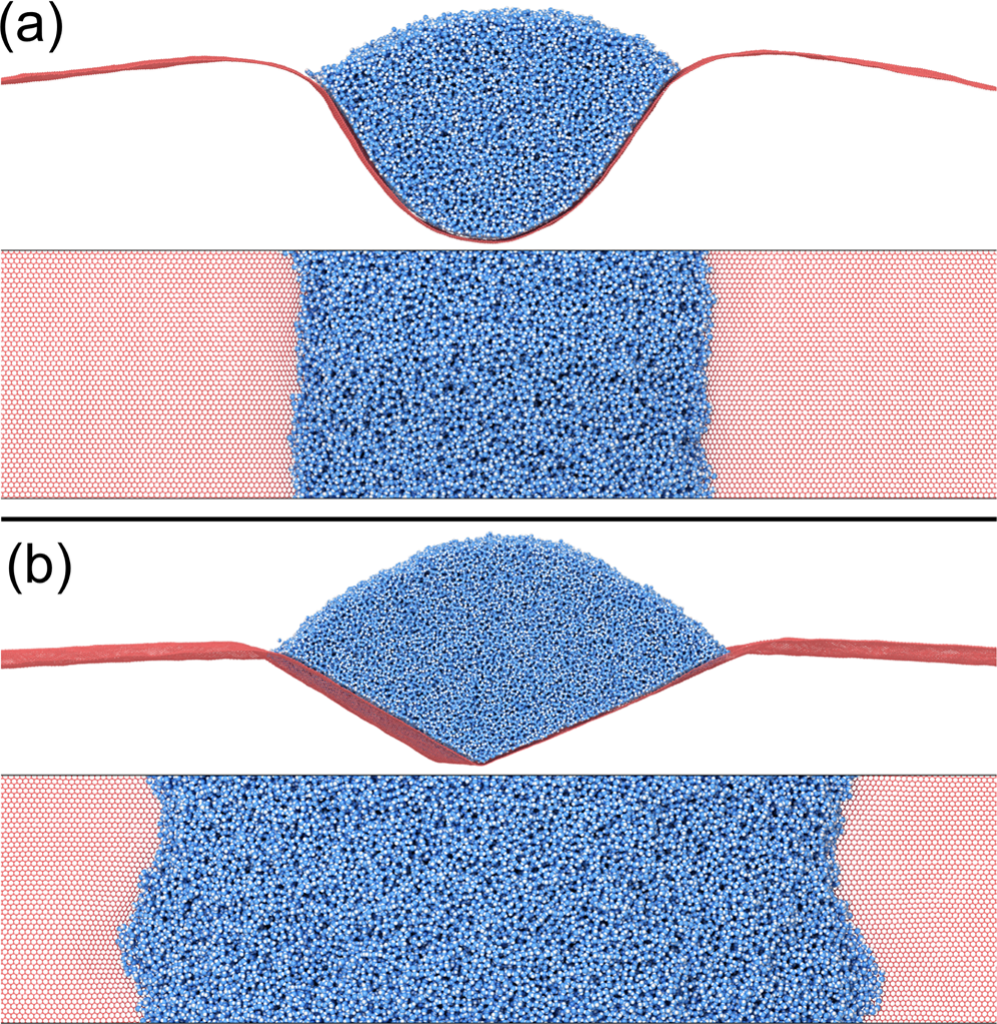
Side and top views of cylindrical droplets over (a) 100 × 12 nm^2^ and (b) 200 × 12 nm^2^ graphene nanoribbons at a low tension. For smooth deformations, as shown in (a), the contact line remains straight. In (b), the sheet has transverse wrinkles and an associated curved contact line. These wrinkles cause a locally very large bending rigidity that prevents the proper equilibrium shape to manifest. Such nonrelaxed samples have been discarded from the analysis in this paper.

The final difference between the continuum theory and MD simulations is that the former is 2D, while the ribbons in our MD simulations are three-dimensional. As we observe a changing wetting angle also when making the ribbons more narrow, we cannot attribute the changes in θ_Y_ to differing dimensionality. On the contrary, in three dimensions, static wrinkles, or creases, can drastically increase the local bending rigidity due nonzero Gaussian curvature. For some of the wider samples, such creases formed during the relaxation. An example is shown in Figure 7b. We attribute this to the onset of hydrodynamic instability, where the cylindrical droplet starts forming necks and bulges before the graphene membrane has folded around it. As the TCL is not straight during this process, transverse tractions appear that wrinkle the membrane. The consequence of this is that the system will not fully relax, resulting in finite values of β and θ_Y_ even when . Hence, this mechanism would push the threshold tension of the vesicle transition to even lower values of , and data points from such metastable states have already been discarded in our analysis.

## Conclusions

In conclusion, using large-scale MD simulations of droplets on suspended graphene nanoribbons under external tension, we have found that existing macroscopic continuum theories work qualitatively well also at the nanoscale. In particular, for large deformations (low tensions), one obtains a good agreement between atomistic simulations and continuum theory provided one allows for a nonconstant θ_Y_. We stress that different sensitivities of the results with respect to Γ and θ_Y_ imply that uncertainties in material parameters cannot account for the observed nonconstant wetting angle. As continuum theory still applies, albeit modified, we expect that for larger systems, one will recover agreement with theories predicting constant wetting angles. However, to further increase system sizes, coarse-grained models will be needed.

While the wetting angle itself is of fundamental interest, our results also have a more direct consequence, which may be easier to detect. This concerns the magnitude of the external tension at which vesicles begin to form. Our simulations and modified model show that this occurs at a tension that can be considerably lower than predicted by macroscopic continuum theory. This is important in situations in which large deformations are of interest. Potential areas of impact are design of nanoscale biomimetic actuators that respond to moisture, capillary origami, vesicle formation, budding, etc. (the latter being useful in, for instance, drug delivery), and other phenomena involving highly flexible membranes.
